# Application of the Sepsis-3 Consensus Criteria in a Geriatric Acute Care Unit: A Prospective Study

**DOI:** 10.3390/jcm8030359

**Published:** 2019-03-13

**Authors:** Davide Bastoni, Andrea Ticinesi, Fulvio Lauretani, Simone Calamai, Maria Letizia Catalano, Pamela Catania, Martina Cecchia, Nicoletta Cerundolo, Claudia Galluzzo, Manuela Giovini, Giulia Mori, Marco Davìd Zani, Antonio Nouvenne, Tiziana Meschi

**Affiliations:** 1 Department of Medicine and Surgery, University of Parma, Via Antonio Gramsci 14, 43126 Parma, Italy; dbastonidoc@gmail.com (D.B.); simonecalamai@hotmail.it (S.C.); marialaetitia2@gmail.com (M.L.C.); pcatania@ao.pr.it (P.C.); cecchia.martina@gmail.com (M.C.); ncerundolo@ao.pr.it (N.C.); claudia88.galluzzo@gmail.com (C.G.); manuelagiovini@gmail.com (M.G.); giuliamori2@gmail.com (G.M.); marcodavid.zani@gmail.com (M.D.Z.); tiziana.meschi@unipr.it (T.M.); 2 Emergency Department, Azienda Ospedaliera di Piacenza, Via Giuseppe Taverna 49, 29121 Piacenza, Italy; 3 Geriatric-Rehabilitation Department, Azienda Ospedaliero-Universitaria di Parma, Via Antonio Gramsci 14, 43126 Parma, Italy; flauretani@ao.pr.it (F.L.); anouvenne@ao.pr.it (A.N.); 4 Post-Graduate Specialization Schoool in Emergency-Urgency Medicine, University of Parma, Via Antonio Gramsci 14, 43126 Parma, Italy

**Keywords:** sepsis, acute infection, comorbidity, frailty, qSOFA, SIRS

## Abstract

The prognostic value of quick Sepsis-related Organ Failure Assessment (qSOFA) score in geriatric patients is uncertain. We aimed to compare qSOFA vs. Systemic Inflammatory Response Syndrome (SIRS) criteria for mortality prediction in older multimorbid subjects, admitted for suspected sepsis in a geriatric ward. We prospectively enrolled 272 patients (aged 83.7 ± 7.4). At admission, qSOFA and SIRS scores were calculated. Mortality was assessed during hospital stay and three months after discharge. The predictive capacity of qSOFA and SIRS was assessed by calculating the Area Under the Receiver Operating Characteristic Curve (AUROC), through pairwise AUROC comparison, and multivariable logistic regression analysis. Both qSOFA and SIRS exhibited a poor prognostic performance (AUROCs 0.676, 95% CI 0.609–0.738, and 0.626, 95% CI 0.558–0.691 for in-hospital mortality; 0.684, 95% CI 0.614–0.748, and 0.596, 95% CI 0.558–0.691 for pooled three-month mortality, respectively). The predictive capacity of qSOFA showed no difference to that of SIRS for in-hospital mortality (difference between AUROCs 0.05, 95% CI −0.05 to 0.14, *p* = 0.31), but was superior for pooled three-month mortality (difference between AUROCs 0.09, 95% CI 0.01–0.17, *p* = 0.029). Multivariable logistic regression analysis, accounting for possible confounders, including frailty, showed that both scores were not associated with in-hospital mortality, although qSOFA, unlike SIRS, was associated with pooled three-month mortality. In conclusion, neither qSOFA nor SIRS at admission were strong predictors of mortality in a geriatric acute-care setting. Traditional geriatric measures of frailty may be more useful for predicting adverse outcomes in this setting.

## 1. Introduction

Sepsis is defined as a life-threatening organ dysfunction, caused by a dysregulated host response to infection [1]. It is associated with prolonged hospital stay, high mortality, and massive economic burden for healthcare systems [2], especially in older people with chronic comorbidities [3]. Previously, the clinical definition of sepsis was based on the Systemic Inflammatory Response Syndrome (SIRS) criteria (temperature >38 °C or <36 °C, heart rate >90, respiratory rate >20, or PaCO_2_ <32 mmHg, white blood cell count >12,000/mm^3^ or <4000/mm^3^), that ultimately proved to not be specific enough for the prediction of complications and mortality [4,5,6,7]. 

In 2016, The Third International Consensus Definitions on Sepsis and Septic Shock (Sepsis-3) revised the definition of sepsis and the clinical approach to patients with suspected infection, according to the risk of in-hospital mortality [1]. Sepsis-3 supported the adoption of the Sequential Organ Failure Assessment (SOFA) score instead of SIRS criteria in patients admitted to intensive care units (ICUs) with suspected infection [1,8,9]. 

For non-ICU patients, a simplified version of SOFA, the quick Sepsis-related Organ Failure Assessment score (qSOFA), was recommended and validated for predicting the onset of septic shock and in-hospital mortality [9]. This tool consists of three items: altered mentation with a Glasgow Coma Scale (GCS) <15, respiratory rate >22, and systolic blood pressure <100 mmHg. Each item can either score 0 (normal finding) or 1 (abnormal finding).

In the validation study, performed in a retrospective group of 66,522 patients admitted to general wards, qSOFA was able to predict in-hospital mortality with a significantly higher accuracy than SIRS criteria [9]. In spite of this, several following studies downsized the clinical relevance of qSOFA, highlighting that its validity for predicting adverse outcomes may be different according to the clinical setting and characteristics of the population [10]. 

In ICUs, the prognostic performance of qSOFA was generally inferior to that of SIRS and SOFA [8]. The use of qSOFA for predicting adverse outcomes in critical patients before ICU admission [11,12] and in groups of emergency department patients with suspected acute bacterial infection [13,14,15,16,17] has given inconclusive results. In adult patients admitted to general medical wards, qSOFA generally proved superior to SIRS for mortality prediction [10,18,19]. However, a recent meta-analysis of 38 studies found that, in a non-ICU population, qSOFA had a poorer sensitivity, but a better specificity, than SIRS for in-hospital mortality [20]. 

To date, very few studies have been focused on geriatric patients. However, these patients represent the largest demographic group at risk of sepsis and sepsis-related adverse outcomes, especially when multimorbidity, frailty, and disability are present. They often have atypical manifestations of acute infections, including delirium, and lack the typical signs and symptoms such as fever, chills, and increased white blood cells [21,22]. Other prognostic parameters not included in qSOFA, such as reduced functional performance, frailty, delirium, and malnutrition, may have a relevant impact on mortality [22,23,24], making prognostic evaluation of geriatric septic patients even more challenging. Frailty and multimorbidity are in fact well-known adverse prognostic factors that can modify the course of any acute illness [25,26]. These considerations raise concerns for the applicability of novel sepsis criteria like the qSOFA scale on older patients admitted to acute geriatric units with suspected infection. 

The aim of this prospective observational study was thus to compare qSOFA scores and SIRS criteria in predicting in-hospital mortality (main outcome) and pooled three-month mortality (secondary outcome), in a group of older multimorbid patients admitted with suspected sepsis to an acute geriatric ward. A secondary aim was also to compare the predictive validity of qSOFA and SIRS with generalized frailty scores for both primary and secondary outcomes. 

## 2. Materials and Methods

### 2.1. Study Setting and Population

At the Internal Medicine and Critical Subacute Care Unit of Parma University Hospital, Italy, a group of older (age ≥65) patients urgently admitted from the emergency department with clinical suspicion of acute bacterial infection were prospectively enrolled from November 2016 to July 2018. The setting of the study was an acute geriatric hospital ward, mainly devoted to the management of acute illnesses of complex multimorbid patients, with a high prevalence of admissions for acute bacterial infections (22%) [27]. 

Patients were enrolled in the study at the moment of ward admission, always within 24 h from ED arrival. Multimorbidity was assessed according to chronic disease count and defined as the presence of at least 2 chronic diseases, as suggested by Barnett et al [28]. Only multimorbid patients were included in the study, since this condition represents an important risk factor for sepsis in the geriatric population [26]. 

The suspicion of bacterial infection was defined as an empirical start for antibiotic therapy (oral or systemic), and sampling of body fluids for cultures was prescribed by the managing physician, according to results from medical history, physical examination, laboratory, and imaging tests. In accordance with the Sepsis-3 recommendations [9], if the antibiotic treatment was started before sampling for cultures, patients were considered for inclusion only if the first antibiotic administration had been performed in the 24 h preceding ward admission and study enrolment. On the contrary, if the sampling for cultures was made before antibiotic administration, the antibiotic should have been started in the following 72 h. 

Patients whose timing of antibiotic administration was not compatible with the above mentioned criteria, together with those who received antibiotic treatment for other conditions in the 30 days before admission, and those who refused or were unable to give written informed consent were excluded. Patients with documented terminal illness (established life expectancy ≤6 months) due to advanced-stage chronic disease or cancer were excluded as well, since they represented a distinct population with an extremely high risk of short-term mortality [29]. 

The sample size was set at 272 patients. Since recent epidemiological data from Italy indicate a mortality of 14.6% during hospitalization for older patients with sepsis [30], the expected in-hospital mortality for the present study was cautiously set at 12.5%. To demonstrate the non-inferiority of qSOFA vs. SIRS for predicting in-hospital mortality in the studied population, we expected an Area Under the Receiver Operating Characteristic (AUROC) curve for qSOFA equal to at least the lower limit of the 95% confidence interval (CI) of the AUROC for SIRS prediction of in-hospital mortality detected in the qSOFA validation study, which was 0.75 [9]. Thus, we assumed an AUROC for qSOFA of 0.75. A sample of 272 participants allowed us to reach a statistical power of 81% to detect a statistically significant difference (*p* < 0.05) of 0.15 between the assumed AUROC of qSOFA and the AUROC of a hypothetical low-performance test (0.6) (calculation performed with PASS-14, NCSS, Kaysville, UT, USA). 

### 2.2. Study Procedures

At the moment of admission in the study ward, vital signs, including arterial pressure, heart rate, respiratory rate, temperature, and oxygen capillary saturation, were measured. A GCS score was calculated during physical examination. Blood cell count and blood gas analysis were prescribed within 24 h of ward admission as part of routine clinical practice for patients with suspected acute infection. These parameters were used for calculating the SIRS and qSOFA scores.

The SIRS score was calculated, assigning zero (absent) or one point (present) to each of the following: Temperature >38 °C or <36 °C, heart rate >90, respiratory rate >20 or PaCO2 <32 mmHg, white blood cell count >12000/mm^3^ or <4000/mm^3^ [1]. The SIRS criteria for sepsis were met if the score was ≥2. The qSOFA score was calculated, assigning zero (absent) or one point (present) to each of the following: GCS <15, respiratory rate >22, systolic blood pressure <100 mmHg [1].

The results of other blood tests of possible prognostic significance for patients with suspected sepsis were also considered, including serum C-reactive protein (CRP), procalcitonin, lactate, creatinine, electrolytes, and parameters of hepatic function. 

In addition, at the time of the patient’s admission to our unit, we calculated:

- The Cumulative Index Rating Scale (CIRS) Comorbidity Score (CIRS-CS) and Severity Index (CIRS-SI), a validated index of multimorbidity and clinical complexity designed for geriatric patients [31], which has been shown to be associated with the risk of acute bacterial infections [32,33]. CIRS-CS was calculated as the sum of the scores (from 0 to 4, where 0 = no disease and 4 = likely lethal disease) assigned to each of the 14 items corresponding to the body’s organs/systems. CIRS-SI was calculated as the number of items with a score ranking 3 or 4;
- The Rockwood Clinical Frailty Scale (RCFS) referred to the patient’s performance before admission. This is a validated score evaluating the presence of frailty or disability in geriatric patients, according to the deficit accumulation model, ranging from 1 (very good physical performance in healthy active aging) to 9 (bedridden patient with short term life expectancy) [34].

During hospital stay, patients were constantly evaluated and any complication or adverse outcome (intensive care unit transferal, death) was assessed. The primary infection site, either assumed according to symptoms and clinical presentation or documented by positive cultures, was collected for each patient. In-hospital mortality was the primary outcome. A follow-up interview by phone was conducted with the patients discharged, or with the caregiver, after three months. The questions investigated mortality, hospital readmissions, and their timing. Pooled three-month mortality was considered as the secondary outcome of the study. 

### 2.3. Statistical Analyses

Data were reported as means and standard deviation (SD) for normally distributed variables, median and interquartile range (IQR) for abnormally distributed variables, or as percentage for discrete variables. Variables were compared between patients who died during hospital stay and survivors by using ANOVA or the Kruskal–Wallis test as appropriate, according to the variable type and distribution. 

Receiver Operating Characteristic (ROC) curves and AUROCs were calculated for qSOFA, SIRS, and RCFS for the prediction of the primary outcome (in-hospital mortality) and secondary outcome (pooled three-month mortality). Pairwise comparison of ROC curves was performed using DeLong et al.’s method [35] with MedCalc software v. 18.11.3 (MedCalc™, 2019). 

Logistic regression models were then used to examine the relationship between baseline qSOFA or SIRS and the primary or secondary outcomes. For each analysis, model 1 tested the univariable association between either qSOFA or SIRS and each of the considered outcomes. In model 2, bivariable logistic regression was performed, adding RCFS to sepsis-specific scores. Then, in model 3, multivariable logistic regression was performed, considering as covariates all the clinical variables that differed between survivors and non-survivors. 

No data were missing for the primary analyses (i.e., qSOFA and SIRS prediction for in-hospital mortality). Follow-up drop-outs (i.e., those patients or caregivers who were unreachable or refused to respond to the phone call three months after discharge) were not considered in the secondary analyses (prediction of pooled three-month mortality). 

All analyses were performed using SAS (version 8.2; SAS Institute Inc., Cary, NC, USA) with a statistical significance level set at *p* < 0.05 and a statistical power of 80%. 

### 2.4. Ethical Statement

All the study procedures were carried out following the latest amendments of the Declaration of Helsinki, and the Good Clinical Practice principles. All participants gave written informed consent. The Ethics Committee of Parma Province approved the study protocol (ID 33332, 9/20/2016).

## 3. Results 

### 3.1. General Characteristics of the Population

The study population was composed of 272 patients, with a mean age of 83.7 ± 7.4. Among these, 247 completed the follow-up, while the remaining 25 were unreachable by phone, or refused to complete the interview. The main characteristics of patients included are summarized in Table 1.

The patients were highly representative of an older population with a high level of comorbidity (CIRS-CS median 14, IQR 10–17), frailty (RCFS median 5, IQR 4–6), and polypharmacy (median number of drugs chronically taken before admission 6, IQR 4–9). The prevalence of dementia and cancer was 34.6% and 12.9%, respectively. 

The etiological diagnosis of infection, with positive body fluid cultures, was reached in only 30.5% of patients. The majority of the infections was assumed to spread from the respiratory tract (57.6%), while 25.8% were from the urinary tract, 11.8% from the gastro-enteric tract, and 4.8% from other sources. The qSOFA score median was 1 (IQR 0–1), and the SIRS score median was 1 (IQR 1–2). The in-hospital mortality recorded in the entire cohort was 11.4%, while, among the 247 participants who completed follow-up, the pooled three-month mortality was 27.5%. A comparison of the baseline characteristics of patients deceased during hospital stay vs. survivors is presented in Table 1. At baseline, patients who died during hospitalization were older, more frail, and cognitively impaired, and had higher values of qSOFA, SIRS, CIRS-SI scores, and serum CRP (Table 1)

### 3.2. qSOFA vs. SIRS for In-Hospital Mortality Prediction

**Figure 1:**
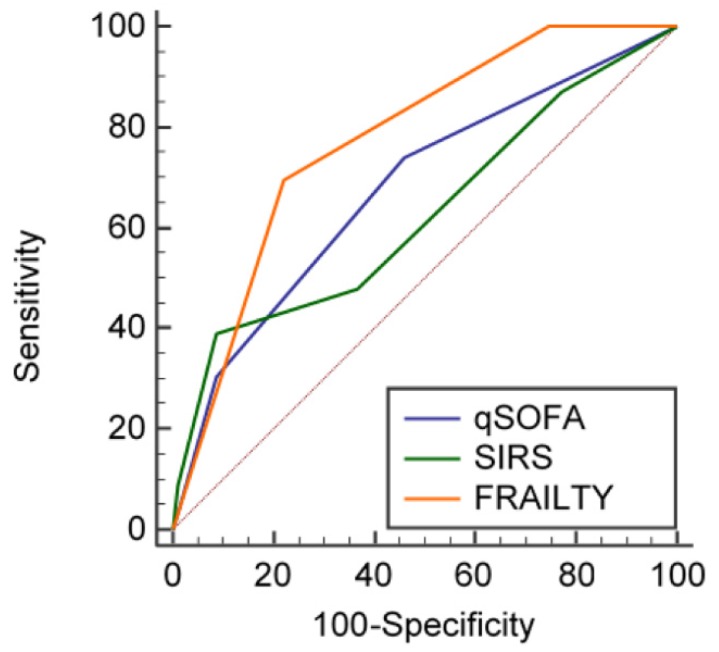
Receiver Operating Characteristic (ROC) curves showing the predictive capacity of quick Sequential Organ Failure Assessment (qSOFA), Systemic Inflammatory Response Syndrome (SIRS), and frailty (operationalized according to the Rockwood Clinical Frailty Scale, RCFS) for in-hospital mortality in the studied geriatric population.

The AUROCs of qSOFA, SIRS, and RCFS for in-hospital mortality were 0.676, 95% CI 0.609–0.738; 0.626, 95% CI 0.558–0.691; and 0.775, 95% CI 0.713–0.829, respectively (Figure 1). Pairwise comparison of ROC curves showed that the predictive capacity of qSOFA against in-hospital mortality was not different to that of SIRS (difference between AUROCS 0.05, 95% CI −0.05 to 0.14, *p* = 0.31) and RCFS (difference between AUROCs 0.09, 95% CI −0.01 to 0.21, *p* = 0.85). A significant difference could however be found between SIRS and RCFS (difference between AUROCs 0.15, 95% CI 0.01–0.30, *p* = 0.04). 

The results of logistic regression models, exploring the association of qSOFA or SIRS with in-hospital mortality, are shown in Table 2 and Table 3. Both qSOFA and SIRS predicted in-hospital mortality only in the univariable models (qSOFA: Odds Ratio (OR) 2.114, 95% CI 1.308–3.419, *p* = 0.002; SIRS: OR 1.703, 95% CI 1.180–2.457, *p* = 0.004), but not in multivariable models adjusted for all possible confounders. 

### 3.3. qSOFA vs. SIRS for Three-Month Mortality Prediction

**Figure 2:**
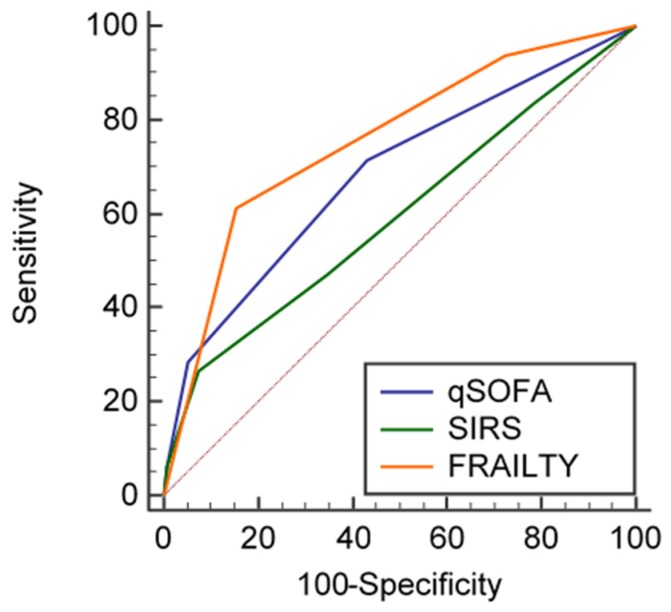
Receiver Operating Characteristic (ROC) curves showing the predictive capacity of qSOFA, SIRS, and frailty (operationalized according to the Rockwood Clinical Frailty Scale) for pooled three-month mortality in the studied geriatric population.

Among the 247 patients who completed follow-up, the AUROCs of qSOFA, SIRS, and RCFS for pooled three-month mortality were 0.684, 95% CI 0.614–0.748; 0.596, 95% CI 0.524–0.665; and 0.756, 95% CI 0.690–0.814, respectively (Figure 2). Pairwise comparison of ROC curves showed that qSOFA had a significantly better performance in predicting three-month mortality than SIRS (difference between AUROCs, 0.09, 95% CI 0.01–0.17, *p* = 0.029). RCFS also performed better than SIRS (difference between AUROCs, 0.16, 95% CI 0.05–0.36, *p* = 0.003), while no difference could be found between ROC curves of qSOFA and RCFS (difference between AUROCs, 0.07, 95% CI −0.01 to 0.15, *p* = 0.09). 

At logistic regression analysis, qSOFA was associated with pooled three-month mortality in all the considered models (OR 2.249, 95% CI 1.009–5.013, *p* = 0.04 at the multivariable model adjusted for all possible confounders) (Table 4). Conversely, the SIRS score was not able to predict pooled three-month mortality when covariates were considered in the multivariable logistic regression models (Table 5).

## 4. Discussion

In a group of older multimorbid patients urgently hospitalized for suspected sepsis, both the qSOFA and SIRS scores, calculated at the moment of admission in a geriatric ward, were poor predictors of in-hospital mortality. However, the qSOFA score resulted a significant and independent predictor of three-month mortality in those who completed follow-up, unlike the SIRS score. In the studied population, the presence of frailty, measured according to the RCFS score, proved a better prognosis predictor than sepsis-specific scales at ROC analysis, but its predictive capacity against in-hospital mortality was not independent of covariates. 

To our knowledge, this is one of the few studies on the novel Sepsis-3 criteria specifically focused on geriatric multimorbid patients, and the first one performed in an acute-care geriatric ward. In fact, previous studies enrolling only subjects aged 70 or older were performed in emergency departments [36,37] or primary care [38]. The results of these studies are apparently in conflict with our findings, since they showed a good prognostic performance by qSOFA for short-term mortality in emergency departments [36,37] and a better prognostic performance by SIRS in primary care [38]. However, recent literature supports the concept that the predictive capacity of qSOFA and SIRS may differ, according to the characteristics of patients and healthcare settings [10,20], and this consideration also applies to older patients, who are admitted to non-ICU wards. 

Geriatric patients with suspected sepsis are complex patients with a very high burden of multimorbidity and frailty. Both of these elements are able to negatively influence prognosis independently of the presence of infections [39,40], and may also reduce the response to treatments [25,26,41]. In this light, it is no surprise that two relatively simple scores, qSOFA and SIRS, were not able to predict short-term adverse outcomes during hospitalization for suspected sepsis. The physio-pathological cascade leading to an adverse prognostic trajectory may in fact include dysregulation of multiple parameters, not included in sepsis-specific scores validated for an adult population without frailty and multimorbidity. One of these parameters may be represented by the inflammatory response that can be monitored during acute infection with serum CRP [42]. In our findings, this single parameter, although extremely non-specific, was able to predict in-hospital mortality. Interestingly, CRP elevation is actively involved in the physio-pathology of frailty and represents a common feature of subjects with multimorbidity [42,43]. 

More comprehensive scores, such as SOFA, may be more suitable for the prognostic evaluation of geriatric patients with acute infections. This assumption was also suggested by Szakmany et al. [44], who found that SOFA, but not qSOFA and SIRS scores, predicted short-term mortality in a population of patients, mostly over 65 years old, admitted to emergency departments or general medical wards for suspected sepsis. 

It is also noteworthy that, even in non-geriatric settings, only a minority of deaths occurring in patients admitted for sepsis are directly and undoubtedly attributable to the infection [45,46]. The attributable fraction of mortality related to sepsis has been esteemed in 15% of patients admitted to ICUs and 24.1% of patients admitted to non-ICU wards [45,46]. In this scenario, frailty and multimorbidity may play a relevant role in defining the prognostic trajectory, even when an acute, disseminated, and life-threatening infection is present. Interestingly, in a nation-wide point-prevalence sepsis study conducted in Wales, Kopzynska et al. [47] found that frailty, measured with the RCFS, was a significant predictor of mortality, unlike qSOFA or SIRS. 

Thus, our findings put into question the clinical usefulness of qSOFA and SIRS scales in multimorbid geriatric patients with suspected sepsis, supporting the use of more traditional tools belonging to comprehensive geriatric assessment for the prognostic evaluation. Some data from the literature also suggest that more complex sepsis-specific scales, such as SOFA, may be useful in multimorbid geriatric patients [44,47], but unfortunately this issue was not investigated in the present study. 

The qSOFA scale may retain some prognostic validity when assessing the risk of mortality three months after discharge, as suggested by the analyses shown in Figure 2 and Table 4. These findings were unexpected, since qSOFA considers a small number of relatively simple physiological derangements, whose impact on the prognosis after hospital discharge may be difficult to explain. However, in the above-mentioned study by Kopczynska et al. [47], conducted in a population with a median age of 73 years old, qSOFA was indeed significantly associated with three-month mortality, although its predictive performance was poor, compared with other more complex tools. Frailty assessment may be much more important than qSOFA for predicting three-month mortality in geriatric septic patients, but the role of qSOFA in this setting may deserve further investigation. 

Our study has some limitations that should be considered. First, the single-center design may limit the applicability of the findings to other groups of geriatric patients, especially in light of the existing literature, suggesting that the prognostic performance of qSOFA and SIRS is highly dependent on the clinical setting [10,20]. Second, qSOFA and SIRS were calculated using data referring to the moment of ward admission, and not to the moment of emergency department arrival, therefore, some of the considered physiological parameters might have been modified by urgent treatments administered before ward admission. However, both scores were validated for use in general medical wards [9], and the aim of the study was just to assess the prognostic performance of the scores in a ward setting. Third, subjects with advanced chronic diseases or cancer, with a very high risk of sepsis and mortality [48], were excluded. Moreover, in most cases, the primary site of infection and etiological agents could not be identified with certainty, making it impossible to stratify participants by infection type. There are in fact data suggesting that qSOFA may have an excellent prognostic capacity, especially in patients with respiratory infections [49,50]. 

Despite this, our study provides very important insights into the prognostic validity and clinical pitfalls of the application of qSOFA and SIRS scores in geriatric in-patients with suspected sepsis. Future studies should confirm these findings, and help to refine the diagnostic and prognostic workup of sepsis in older subjects. 

## 5. Conclusions

In a group of older multimorbid patients admitted to an acute geriatric ward for suspected sepsis, both qSOFA and SIRS scores were not able to predict in-hospital mortality. Unlike SIRS, qSOFA seemed to be able to predict mortality three months after discharge. For both outcomes, frailty assessment seemed superior in the prognostic evaluation. The applicability and prognostic validity of Sepsis-3 criteria in the geriatric population is limited, and should be targeted by larger studies in the future. 

## Figures and Tables

**Table 1:** Overview of the main characteristics of the studied population of 272 elderly subjects admitted to an acute geriatric hospital ward with suspected sepsis. A comparison of baseline characteristics between patients discharged alive (*n* = 241) and patients deceased during hospital stay (*n* = 31) is also shown.

Parameters	Overall Population (*n* = 272)	Survivors at Discharge (*n* = 241)	Dead during Hospital Stay (*n* = 31)	*p* *
Females, *n* (%)	144 (52.9)	126 (52.3)	18 (58.1)	0.54
Age, years	83.7 ± 7.4	83.3 ± 7.3	86.3 ± 7.1	**0.04**
Institutionalized, *n* (%)	44 (16.2)	31 (12.8)	13 (41.9)	**<0.001**
Frailty (RCFS score 4–5), *n* (%)	109 (40.1)	102 (42.3)	7 (22.5)	**<0.001**
Disability (RCFS score >5), *n* (%)	123 (45.2)	99 (41.1)	24 (77.4)	
RCFS, score	5 [4–6]	5 [4–6]	7 [6–8]	**<0.001**
Cancer, *n* (%)	35 (12.9)	30 (12.5)	5 (16.1)	0.56
Dementia, *n* (%)	94 (34.6)	76 (31.5)	18 (58.1)	**0.004**
CIRS-CS, score	14 [10–17]	13 [10–17]	14 [13–16]	0.88
CIRS-SI, score	2 [2–3]	2 [2–3]	3 [2–3]	**<0.001**
Drugs taken before admission, *n*	6.2 ± 3.1	6.1 ± 3.1	7.3 ± 3.0	0.06
Pulmonary infection, *n* (%)	156 (57.6)	141 (58.5)	15 (48.3)	0.40
Gastrointestinal infection, *n* (%)	32 (11.8)	32 (13.3)	1 (3.2)	
Urinary tract infection, *n* (%)	70 (25.8)	55 (22.8)	15 (48.4)	
Other primary infection, *n* (%)	13 (4.8)	13 (5.4)	0 (0)	
qSOFA, score	1 [0–1]	0 [0–1]	1 [0–2]	**<0.001**
SIRS, score	1 [1–2]	1 [1–2]	2 [1–3]	**0.003**
C-reactive protein, mg/L	109 [61–182]	104 [57–170]	194 [96–250]	**<0.001**
Body temperature, °C	37.1 ± 0.7	37.1 ± 0.7	37.1 ± 0.5	0.70
Heart rate, bpm	85.9 ± 17.8	84.6 ± 17.2	96.8 ± 18.9	**0.08**
Systolic blood pressure, mmHg	127.4 ± 21.9	127.8 ± 21.2	123.5 ± 26.8	0.31
Diastolic blood pressure, mmHg	72.5 ± 10.6	72.8 ± 10.2	70.9 ± 13.7	0.38
Peripheral oxygen saturation, %	95.0 ± 2.9	95.2 ± 2.7	94.3 ± 3.8	0.11
Respiratory rate, bpm	19.3 ± 5.1	19.2 ± 4.9	20.3 ± 6.9	0.28
GCS, score	14.6 ± 1.5	14.7 ± 1.3	13.8 ± 2.7	**0.001**
PaCO2, mmHg	38.3 ± 11.2	37.3 ± 8.8	45.1 ± 20.4	**0.004**
WBC count, n/mm ×1000	9.8 [6.5–13.9]	9.6 [6.6–13.9]	11.6 [6.4–15.4]	0.59
Procalcitonin, ng/mL	0.7 [0.1–3.0]	0.5 [0.1–2.7]	2 [0.9–9.6]	0.72

* *p* values < 0.05 are indicated in bold. Data presented as mean ± standard deviation, median [interquartile range], or number and (percentage) as appropriate, according to the type of variable and normality of distribution. Comparisons performed with ANOVA and Kruskal–Wallis test, as appropriate. RCFS = Rockwood Clinical Frailty Scale; CIRS-CS = Cumulative Illness Rating Scale Comorbidity Score; CIRS-SI = Cumulative Illness Rating Scale Severity Index; qSOFA = quick Sequential Organ Failure Assessment; SIRS = Systemic Inflammatory Response Syndrome; GCS = Glasgow Coma Scale; WBC = White Blood Cell.

**Table 2:** Logistic regression analysis showing the association of qSOFA score at admission with the primary study outcome (in-hospital mortality) in elderly patients with suspected sepsis (*n* = 272).

Parameters	OR (95% CI)	*p* *
Model 1—Univariable		
qSOFA score	2.114 (1.308–3.419)	**<0.001**
Model 2—Bivariable		
qSOFA score	1.454 (0.801–2.637)	0.21
Frailty	5.242 (2.101–13.083)	**<0.001**
Model 3—Multivariable		
qSOFA score	0.828 (0.245–2.796)	0.76
Frailty	2.534 (0.523–12.270)	0.24
Age	1.005 (0.905–1.116)	0.93
Sex (female vs. male)	0.594 (0.138–2.552)	0.44
Institutionalization	1.928 (0.324–11.461)	0.47
Dementia	0.347 (0.050–2.401)	0.28
CIRS-SI	0.991 (0.480–2.048)	0.98
CRP	1.010 (1.001–1.020)	**0.045**
PaCO_2_	1.041 (0.986–1.100)	0.14

* *p* values < 0.05 are indicated in bold. The multivariable model was built considering the parameters to be significantly different between survivors and non-survivors at descriptive analysis. Frailty was considered as a categorical variable, based on the Rockwood Clinical Frailty Scale score (1–3 = fit; 4–5 = frail; 6–9 = disabled). OR = Odds Ratio; qSOFA = quick Sequential Organ Failure Assessment; CIRS-SI = Cumulative Illness Rating Scale Severity Index; CRP = C-Reactive Protein.

**Table 3:** Logistic regression analysis showing the association of SIRS score at admission with the primary study outcome (in-hospital mortality) in elderly patients with suspected sepsis (*n* = 272).

Parameters	OR (95% CI)	*p* *
Model 1—Univariable		
SIRS score	1.703 (1.180–2.457)	**<0.001**
Model 2—Bivariable		
SIRS score	1.438 (0.932–2.219)	0.10
Frailty	5.710 (2.372–13.744)	**<0.001**
Model 3—Multivariable		
SIRS score	1.084 (0.542–2.168)	0.82
Frailty	2.629 (0.533–12.968)	0.23
Age	1.004 (0.903–1.115)	0.95
Sex (female vs. male)	0.583 (0.136–2.502)	0.47
Institutionalization	1.766 (0.330–9.436)	0.51
Dementia	0.379 (0.058–2.473)	0.32
CIRS-SI	0.984 (0.477–2.030)	0.97
CRP	1.009 (1.001–1.019)	**0.041**
PaCO_2_	1.042 (0.987–1.099)	0.14

* *p* values < 0.05 are indicated in bold. The multivariable model was built considering the parameters significantly different between survivors and non-survivors at descriptive analysis. Frailty was considered as a categorical variable based on the Rockwood Clinical Frailty Scale score (1–3 = fit; 4–5 = frail; 6–9 = disabled). OR = Odds Ratio; SIRS = Systemic Inflammatory Response Syndrome; CIRS-SI = Cumulative Illness Rating Scale Severity Index; CRP = C-Reactive Protein.

**Table 4:** Logistic regression analysis showing the association of qSOFA score at admission with the secondary study outcome (pooled mortality three months after discharge) in elderly patients with suspected sepsis who completed the study follow-up (*n* = 247).

Parameters	OR (95% CI)	*p* *
Model 1—Univariable		
qSOFA score	2.255 (1.510–3.368)	**<0.001**
Model 2—Bivariable		
qSOFA score	1.915 (1.151–3.188)	**0.012**
Frailty	3.958 (2.131–7.352)	**<0.001**
Model 3—Multivariable		
qSOFA score	2.249 (1.009–5.013)	**0.04**
Frailty	2.336 (1.002–5.729)	**0.04**
Age	0.992 (0.915–1.077)	0.85
Sex (female vs. male)	0.369 (0.128–1.066)	0.07
Institutionalization	0.550 (0.135–2.236)	0.40
Dementia	1.474 (0.393–5.528)	0.56
CIRS-SI	1.669 (0.979–2.847)	0.06
CRP	0.998 (0.991–1.005)	0.56
PaCO_2_	1.032 (0.987–1.078)	0.16

* *p* values < 0.05 are indicated in bold. The multivariable model was built considering the parameters to be significantly different between survivors and non-survivors at descriptive analysis. Frailty was considered as a categorical variable based on the Rockwood Clinical Frailty Scale score (1–3 = fit; 4–5 = frail; 6–9 = disabled). OR = Odds Ratio; qSOFA = quick Sequential Organ Failure Assessment; CIRS-SI = Cumulative Illness Rating Scale Severity Index; CRP = C-Reactive Protein.

**Table 5:** Logistic regression analysis showing the association of SIRS score at admission with the secondary study outcome (pooled mortality three months after discharge) in elderly patients with suspected sepsis who completed the study follow-up (*n* = 247).

Parameters	OR (95% CI)	*p* *
Model 1—Univariable		
SIRS score	1.369 (1.032–1.816)	**0.03**
Model 2—Bivariable		
SIRS score	1.260 (0.876–1.812)	0.21
Frailty	4.855 (2.632–8.954)	**<0.001**
Model 3—Multivariable		
SIRS score	1.149 (0.696–1.897)	0.57
Frailty	2.505 (1.038–6.044)	**0.04**
Age	0.995 (0.920–1.077)	0.91
Sex (female vs. male)	0.414 (0.149–1.148)	0.09
Institutionalization	0.693 (0.180–2.665)	0.59
Dementia	1.105 (0.319–3.829)	0.88
CIRS-SI	1.543 (0.930–2.562)	0.09
CRP	0.999 (0.992–1.005)	0.68
PaCO_2_	1.011 (0.969–1.033)	0.22

* *p* values < 0.05 are indicated in bold. The multivariable model was built considering the parameters to be significantly different between survivors and non-survivors at descriptive analysis. Frailty was considered as a categorical variable based on the Rockwood Clinical Frailty Scale score (1–3 = fit; 4–5 = frail; 6–9 = disabled). OR = Odds Ratio; SIRS = Systemic Inflammatory Response Syndrome; CIRS-SI = Cumulative Illness Rating Scale Severity Index; CRP = C-Reactive Protein.
